# Cryptotanshinone inhibits proliferation and induces apoptosis of breast cancer MCF-7 cells via GPER mediated PI3K/AKT signaling pathway

**DOI:** 10.1371/journal.pone.0262389

**Published:** 2022-01-21

**Authors:** Danning Shi, Hongbo Li, Zeye Zhang, Yueshuang He, Meng Chen, Liping Sun, Piwen Zhao

**Affiliations:** 1 School of Life Sciences, Beijing University of Chinese Medicine, Beijing, 100029, China; 2 Department of Gynecology, Affiliated Hospital of Shaanxi University of Chinese Medicine, Shaanxi, 712000, China; 3 School of Traditional Chinese Medicine, Beijing University of Chinese Medicine, Beijing, 100029, China; Columbia University, UNITED STATES

## Abstract

G protein-coupled estrogen receptor (GPER) was reported to be a potential target in the breast cancer therapy. This study aimed to illuminate the function of GPER and its mediated PI3K/AKT pathway in cryptotanshinone (CPT) inducing cell apoptosis and antiproliferation effect on GPER positive breast cancer MCF-7 cells. Cell proliferation was tested by MTT assay. Apoptosis rates were tested by Annexin V-FITC/PI double staining and the cell cycle was researched by flow cytometry. Autodock vina was applied to make molecular docking between CPT or estradiol and GPER. siRNA technique and GPER specific agonist G-1 or antagonist G-15 were applied to verify the mediated function of GPER. Apoptosis and cell cycle related proteins, as well as the key proteins on PI3K/AKT signaling pathway were detected by western blot. The results indicated that CPT could exert antiproliferation effects by arresting cell cycle in G2/M phase and downregulating the expression of cyclin D, cyclin B and cyclin A. Besides, apoptosis induced by CPT was observed. CPT might be a novel GPER binding compounds. Significantly, suppression of PI3K/AKT signal transduction by CPT was further increased by G-1 and decreased by G-15. The study revealed that the effect of antiproliferation and apoptosis treating with CPT on MCF-7 cells might be through the downregulation of PI3K/AKT pathway mediated by activated GPER.

## Introduction

Breast cancer is one of the most priority concerns for women around the world and results in relatively high rates of morbidity and mortality [1]. Globally, it is estimated that more than 1 million people are diagnosed with breast cancer each year and that at least 400,000 women will die from this disease annually, accounting for 14 per cent of all cancer deaths [2,3]. According to the China National Cancer Center, the incidence of breast cancer has obviously increased more than twice as fast as global rates since 1990s, especially in urban city [4,5]. It has long been known that predominantly 17β-estradiol (E2) and its receptors play a crucial role in development of breast cancer. For decades, drugs that targeting the estrogen receptors (ER) through the selective estrogen receptor modulators (SERMs) have been used for treatment of ER positive breast cancer [6,7]. However, primary or acquired drug resistance becomes a major obstacle to hormone therapy, suggesting more complex receptors and signaling pathways involved in breast cancer progression [8]. Therefore, seeking a safe and effective alternative treatment is particularly necessary.

Traditional Chinese medicine (TCM) has attracted much attention because of its natural properties and multi-target functions. *Salvia miltiorrhiza* (Danshen) is one of the most widely used herbal medicines in TCM. Danshen was first used as a treatment for cardiovascular disease. In recent years, researchers have found that it also has significant anti-tumor effects. Especially in the study of its active components, anti-tumor activity was proven in vivo and in vitro [9,10]. Cryptotanshinone (CPT), a diterpene quinone extracted from Danshen, showed inhibition effect in some kinds of tumor cells in vitro, including breast cancer [11–15]. However, the specific mechanism of its pharmacological effects, crucially the molecular signal transduction involved still remains unclear and need to be further research. Besides, the phytoestrogen-like activity of CPT is also expected due to the similarity of its structure with estradiol.

Studies have shown that phytoestrogens are diphenols or polyphenols derived from plants, which can exert the estrogenic or anti-estrogenic through binding with ER [16,17]. G protein coupled estrogen receptor (GPER), a seven transmembrane domain protein, was recognized as a new kind of membrane estrogen receptor which could induce rapid cellular effects [18,19]. Accumulating evidence confirmed that GPER and its mediated signaling pathways are relevant to breast cancer [20,21], however, its role in breast cancer cells has been controversial. Studies indicated that the expression of GPER was positively associated with the development of breast cancer [22]. But nevertheless, number of researches revealed that activation of GPER could suppress the proliferation of breast cancer cells in various methods [23–26]. Although the mechanism of mediation through GPER need to be further explained, GPER might be a novel target for the treatment of breast cancer. Among the pathways mediated by GPER, PI3K/AKT signaling pathway takes an essential part in the proliferation of breast cancer cells, and constitutive activation of it is realized to be crucial in the progression of human breast tumors [27,28]. Since that, suppression of PI3K/AKT function may be a key to cancer therapy.

In this study, we will detect the effects of CPT, especially the molecular mechanisms mediated by GPER and its regulated PI3K/AKT signaling pathway through a series of in vitro experiments in the GPER positive breast cancer MCF-7 cells. CPT as a potential anti-breast cancer natural compound via the mediation of GPER was expected.

## Methods

### Reagents

Cryptotanshione (CPT) obtained from National Institutes for Food and Drug Control (Beijing, China) was prepared into a 10 mM stock solution dissolved by DMSO and stored at -20°C for later use. MTT (KeyGEN BioTECH, Jiangsu, China), DMEM-High Glucose medium (Hyclone company), Fetal bovine serum (Corning Cell Gro, Australia), 0.25% trypsin (Gibco, ThermoFisher Scinetific Inc, USA). Lipofectamine^TM^ 2000 Reagent was from Invitrogen Inc, USA. GPER specific agonist G-1 and antagonist G-15 were obtained from Cayman Chemical (Michigan, USA). The primary antibodies were listed below: cyclin D, cyclin B and cyclin A, Bax, Bcl-2, caspase-3, PI3K (p85) and GPER (Abcam, USA), p-AKT (Ser473, Cell Signaling Technology, USA). GPER siRNA, non-target siRNA and PI3K inhibitor LY294002 were purchased from Santa Cruz Biotechnology, USA.

### Cell culture

Human breast cancer MCF-7 cells (National Infrastructure of Cell Line Resource, Beijing, China) were cultured in DMEM-High Glucose medium supplemented with 10% fetal bovine serum. Besides, 100 μg/ml streptomycin as well as 100 U/ml penicillin was added against contamination and cells were cultured at 37°C in a 5% CO_2_ atmosphere.

### Cell proliferation assay after CPT treatment

Then treated cells with CPT at the concentration of 1, 2.5, 5 and 10 μM respectively for another 24 h or 48 h. 0.2% DMSO was as a control. After administration, 15 μL MTT (1 mg/ml) was added and incubated for 4 h avoiding the impact of light. Removed the supernatants and lysed the cells in 150 μl DMSO. The optical density values were tested at a measuring wavelength of 490nm using a plate reader. Repeated the independent experience three times.

### Cell cycle distribution analysis

Chose, counted and seeded MCF-7 cells in logarithmic growth phase into 6-well plates at a density of 5×10^5^ cells per well. After adhesion, DMEM-high glucose medium was changed with 0, 5, and 10 μM CPT for another 48 h. Then treated MCF-7 cells with 0.25% trypsin and collected into centrifuge tubes. Washed cells with 4°C pre-cooled phosphate-buffered saline (PBS), centrifuged for 1–2 times. 1 mL 70% ethanol was added to fix the cell cycle and cultured overnight at 4°C. Samples were then taken into room temperature and washed with PBS for 1–2 times. 100 μg/mL RNase diluted in PBS was added and maintained at 37°C for 30 min. Stained with 50 μg/ml PI (Sigma, USA) avoiding light. Cell cycle distribution was performed by Flow cytometry (FCM, BD Inc, USA) within 1 h. Repeated the independent experiment for three times.

### Apoptosis rate analysis

Seeded cells in 6-well plates at the density of 5×10^5^ per well and cultured overnight for adhesion. Change the medium with 0, 5 and 10 μM CPT for 48 h. Collected cells and add 500 μL binding buffer to get single-cell suspension. Each group was stained using 5μL Annexin V-FITC for 10 min and then, 5μL PI for 5 min avoiding light at room temperature. Within 1h, apoptosis was determined by FCM. Independent experiment was repeated for three times.

### Molecular docking

In order to further verify the interaction between active components of CPT and GPER, computer molecular docking technology was carried out. The encoding sequence for GPER was retrieved from the Protein data bank (PDB) database and then submitted to the GPCR-I-TASSER (version 5.1), which is an algorithm specifically designed to model G protein-coupled receptors [29]. The resulting conformation was a seven-helix structure. Subsequently, the crystal structure of GPER obtained from GPCR-I-TASSER was optimized by Pymol and Autodock tool software for the later docking. The structure of CPT and estradiol were built by ChemDraw and Chem3D based on their chemical structure respectively. A flexible docking was carried out by Autodock vina, and the grid box was covered on the protein to make a blind docking. Visualization of docking results was built using Pymol and Discovery Studio.

### Small interfering RNA (siRNA) transfection

MCF-7 cells in logarithmic growth phase were selected, counted and seeded into 6-well plates at a density of 5×10^5^ cells per well. When the density reached about 90%, 33 nM GPER-siRNA or non-target siRNA was transfected respectively in MCF-7 cells using Lipofectamine^TM^ 2000 Reagent according to the manufacture instruction. After 48 h, cells were collected for testing the expression of GPER by western blot. Meanwhile, cells with low expression of GPER then treated with CPT for another 48 h, cell viability assay was further applied to investigate the effect of GPER on the antiproliferation of CPT in MCF-7 cells.

### Western blot analysis

Cells were counted and seeded in 6-well plates overnight to keep the cells adhered. The MCF-7 cells were then incubated with CPT in different concentration for 48 h. GPER agonist G-1 or antagonist G-15 was added with 5 μM CPT respectively. Total protein was collected by RIPA lysis buffer on the ice, centrifuging at 12000 rpm for 5 min at 4°C. The supernatant was then collected for measurement by bicinchoninic acid (BCA) protein assay kit (Solarbio, Beijing, China). Samples were firstly separated by 10% SDS-PAGE and then, transferred to PVDF membranes by half-wet transfer on the ice. Primary antibody dilutions were listed blew: cyclin D (1:10,000), cyclin B (1:10,000), cyclin A (1:1000), PI3K (p85, 1:1000), AKT (1:2000), p-AKT (1:2000), GPER (1:250), Bcl-2 (1:2000), Bax (1:100), Caspase-3 (1:1000) and anti β-actin (1:10,000). After incubation at 4°C overnight, the corresponding secondary antibodies was applied. Membranes incubated by ECL luminescent solution were then imaged through the multifunctional molecular imaging system (Azure C-Series C600, USA). Results were analyzed by Image J software (version 1.48, National Institutes of Health, USA). The independent experiment was repeated three times.

### Statistical analysis

Data was analyzed using SPSS 20.0. Results of three independent tests were presented as mean ± S.D. Comparisons among multiple groups were performed by one-way ANOVA. *P* < 0.05 were considered significant.

## Results

### CPT inhibited the viability of MCF-7 cells through G2 phase arrest and apoptosis induction

**Fig 1:**
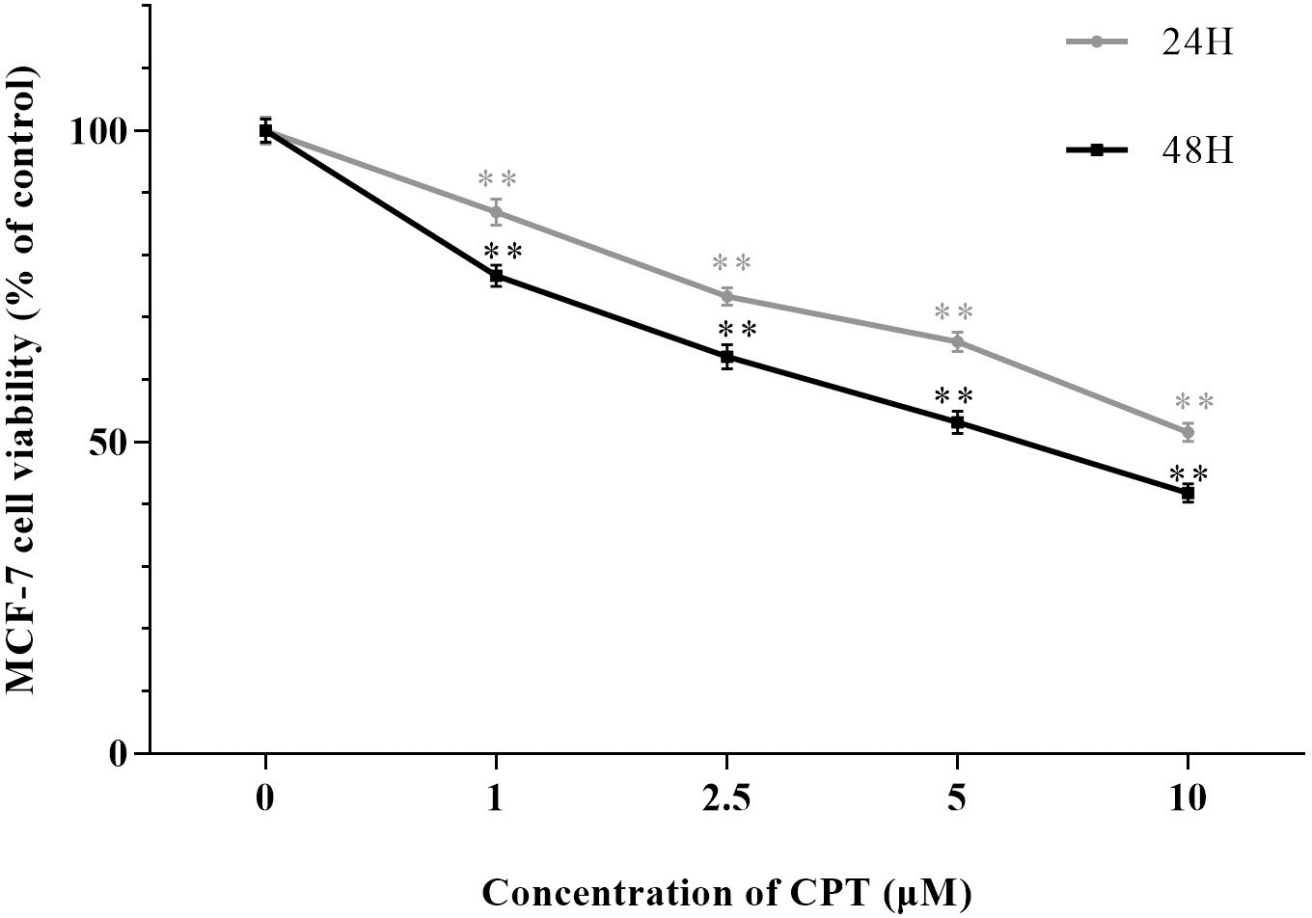
Inhibitory effect of CPT on MCF-7 cell viability. MCF-7 cells were treated by CPT of indicated concentrations for 24 h or 48 h. The results are expressed as $\bar{x}$ ± S.D. ^**^P < 0.01 compared with control group were recognized as statistically significant.

**Fig 2:**
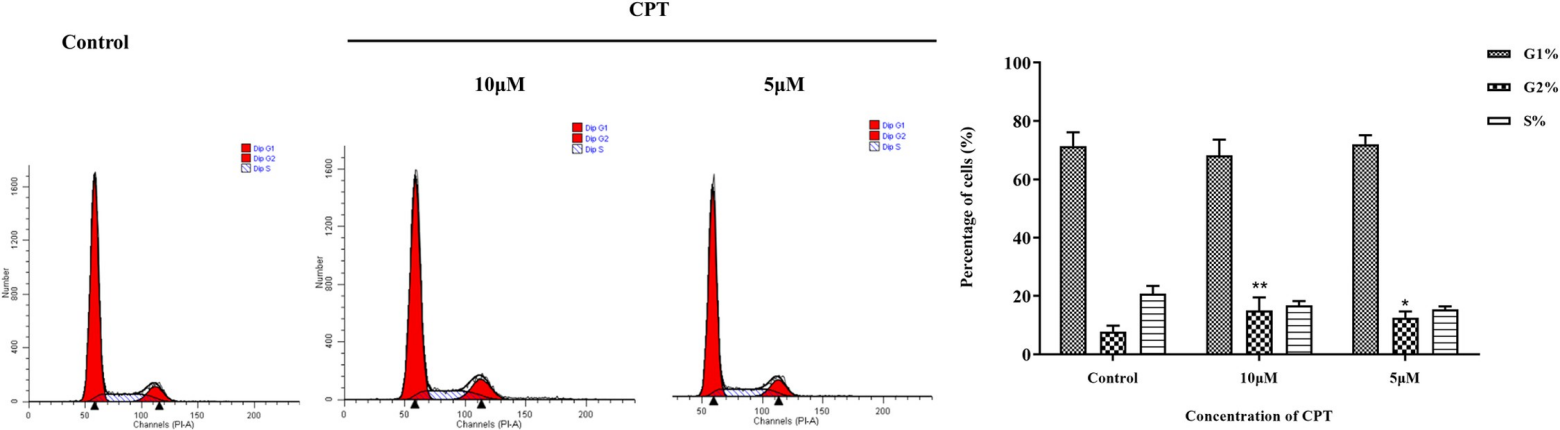
Cell cycle distribution in MCF-7 cells after CPT treatment. The results are expressed as $\bar{x}$ ± S.D. ^*^P < 0.05 or ^**^P < 0.01 compared with control group were recognized as statistically significant.

**Fig 3:**
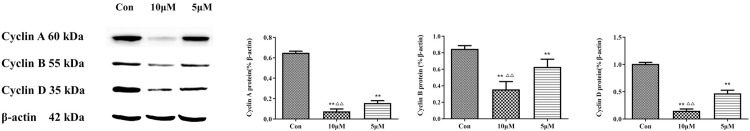
The expression of cyclins in MCF-7 treated by CPT. The results are expressed as $\bar{x}$ ± S.D. ^**^P < 0.01 compared with control group and ^*△△*^*P* < 0.01 compared with 5 μM CPT group were recognized as statistically significant.

**Fig 4:**
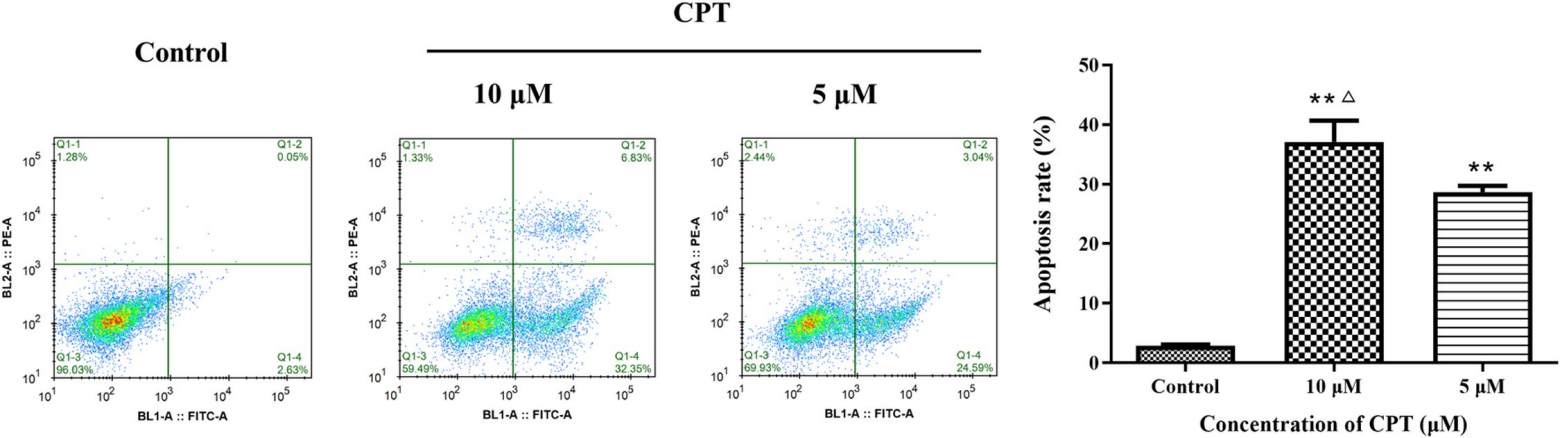
Apoptosis rate tested by Annexin V-FITC/PI double staining. The results are expressed as $\bar{x}$ ± S.D. ^**^P < 0.01 compared with control group and ^*△*^*P* < 0.05 compared with 5 μM CPT group were recognized as statistically significant.

**Fig 5:**
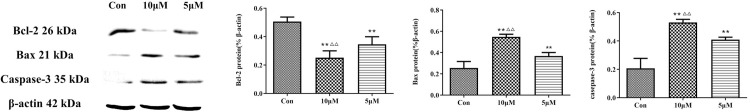
The expression of apoptosis related protein in MCF-7 after treating by CPT. The results are expressed as $\bar{x}$ ± S.D. ^**^P < 0.01 compared with control group and ^*△△*^*P* < 0.01 compared with 5 μM CPT group were recognized as statistically significant.

After Treating by indicated concentrations of CPT (1 μM, 2.5 μM, 5μM and 10 μM) for 24 h and 48 h leaded to 86.9% and 76.7%, 73.4% and 63.7%, 66.1% and 53.2%, 51.6% and 41.8% cell viability compared with control group, respectively (Fig 1). Significantly, we detected that CPT could inhibit the viability of MCF-7 cells in a dose and time dependent manner. The effect of CPT on cell cycle progression analyzed by FCM was exhibited in Fig 2. An increasing of the percentage of MCF-7 cells in G2-phase to 12.6 ± 2.1% and 15.0 ± 4.5% was observed using 5 μM CPT and 10 μM CPT, compared with the control group, 7.8 ± 2.0%. The results illustrated that antiproliferative effect on MCF-7 cells induced by CPT might be relevant to G2-phase arrest. Moreover, the expression of cyclin D, cyclin B and cyclin A tested by western blot indicated that CPT have an inhibitory effect on cyclins. As shown in Fig 3, the decreased expression of cyclin B was relatively attenuate compared with cyclin A and cyclin D. Furthermore, Apoptosis rate was increased from 28.7% to 37.1% by using 5 to 10 μM CPT, significantly compared with the 2.9% for control (Fig 4). In addition, the expression of apoptosis related protein Bax and caspase-3 were increased, however, anti-apoptosis related protein Bcl-2 was inhibited after treating by 5 and 10 μM CPT (Fig 5).

### Identification of CPT as a novel GPER modulator

**Fig 6:**
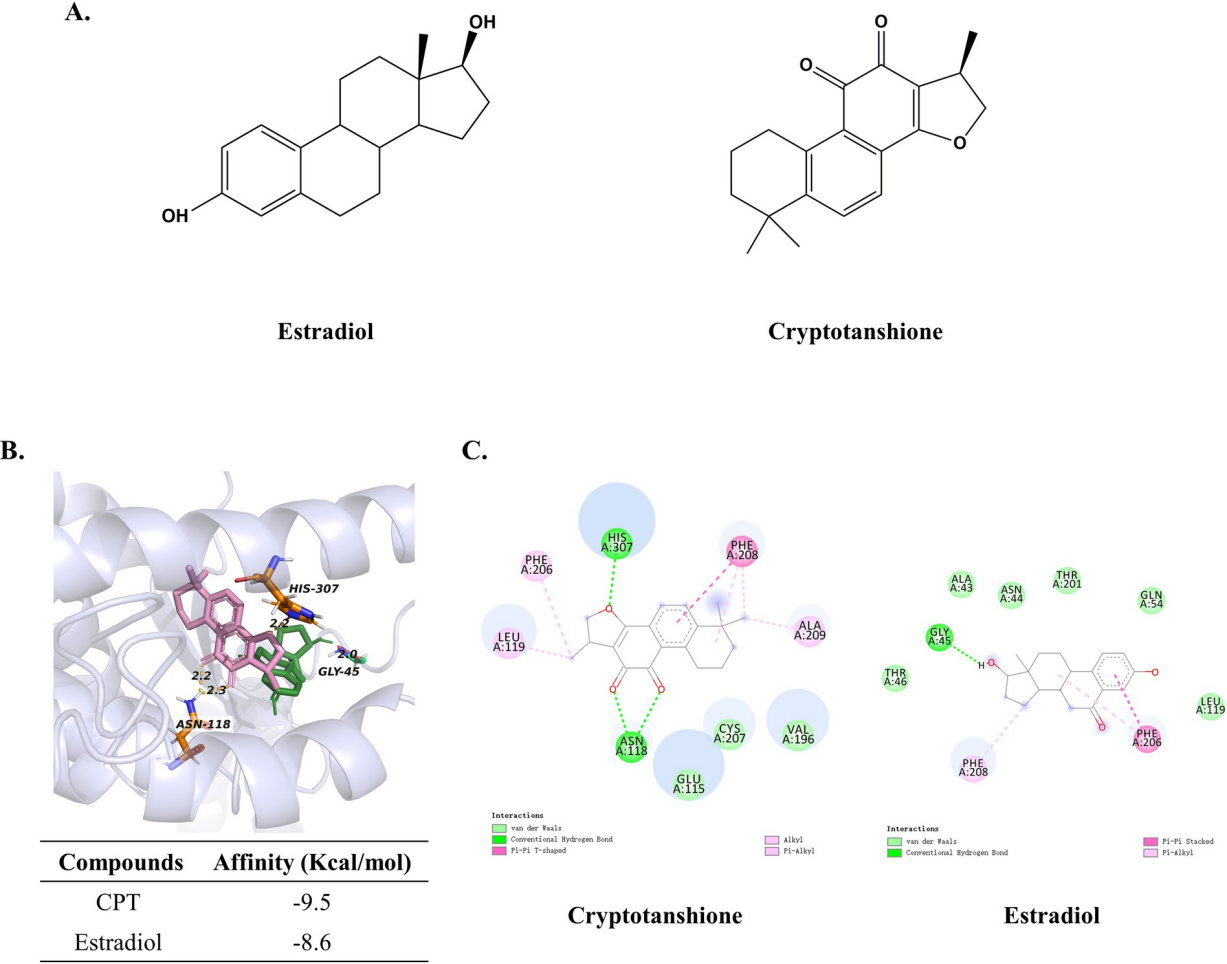
Molecular docking between GPER and CPT or estradiol. (A) Chemical structure of CPT and estradiol. (B) Molecular model of estradiol and CPT docking with GPER. The protein structure is shown in purple (ribbon), CPT and estradiol are shown in pink and green carbon scheme, respectively. The yellow lines indicated the hydrogen bond between CPT and GPER, the red line indicated the hydrogen bond between estradiol and GPER. (C) Bidimensional docking results. Bubbles represent amino acid residue. Meaning of lines in different color was shown on the figures.

Since the chemical structure of estradiol is similar to CPT with 3 cyclohexane rings, one cyclopentane ring and 17 carbon atoms (Fig 6A), estradiol was chosen for the simulations as comparative. The affinity of CPT-GPER was -9.5 kcal/mol, compared with the -8.6 of estradiol-GPER. CPT has interaction energy comparable to that of estradiol with GPER. The results of visualization revealed that CPT could bind with GPER on amino acid residues known as ASN-188 and HIS-307 in the form of conventional hydrogen bond (Fig 6B), besides, pi-pi accumulation with PHE-208, van der Waals and other interactions promoted the combination tightly. For the estradiol, we observed it could be interacted with the hydrogen bond of the amino residue on GLY-45, and pi-pi stacked on PHE-206, pi-alkyl on PHE-208 (Fig 6B and 6C).

### The effect of CPT on MCF-7 cells could be mediated by GPER

**Fig 7:**
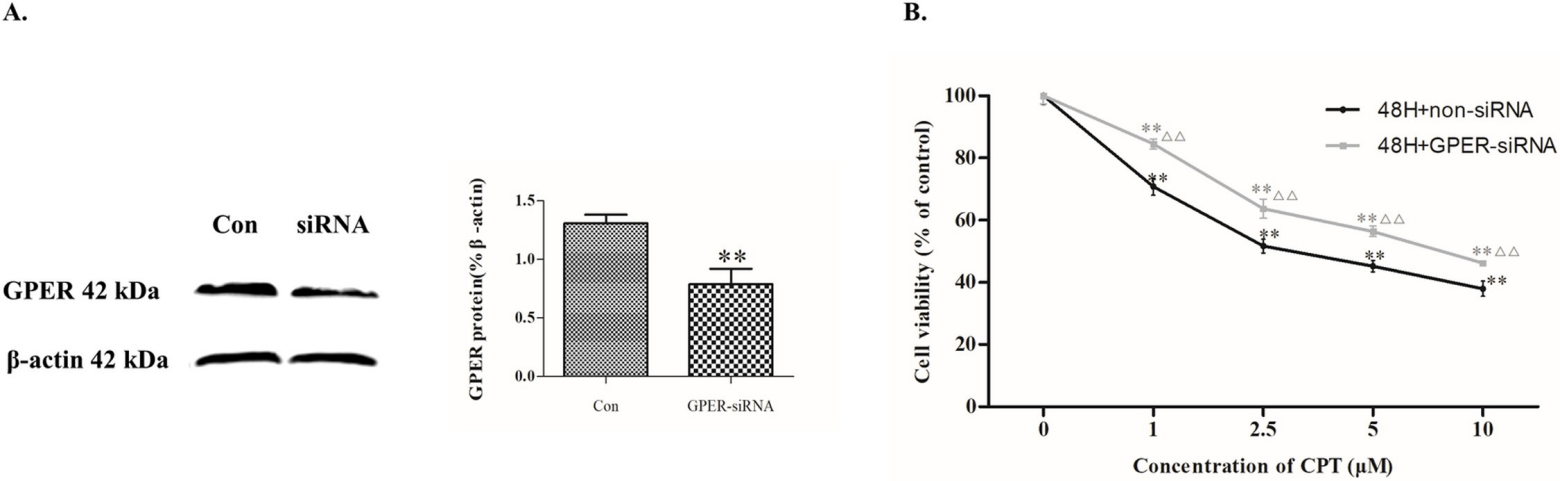
CPT inhibits MCF-7 cell proliferation mediated by GPER. (A) The expression of GPER. **P < 0.01 compared with control group. [B] Viability of MCF-7 cells with knockdown of GPER. The results are expressed as $\bar{x}$ ± S.D. ^**^P<0.01 compared with control group or ^△△^*P* < 0.01 compared with non-siRNA group were recognized as statistically significant.

**Fig 8:**
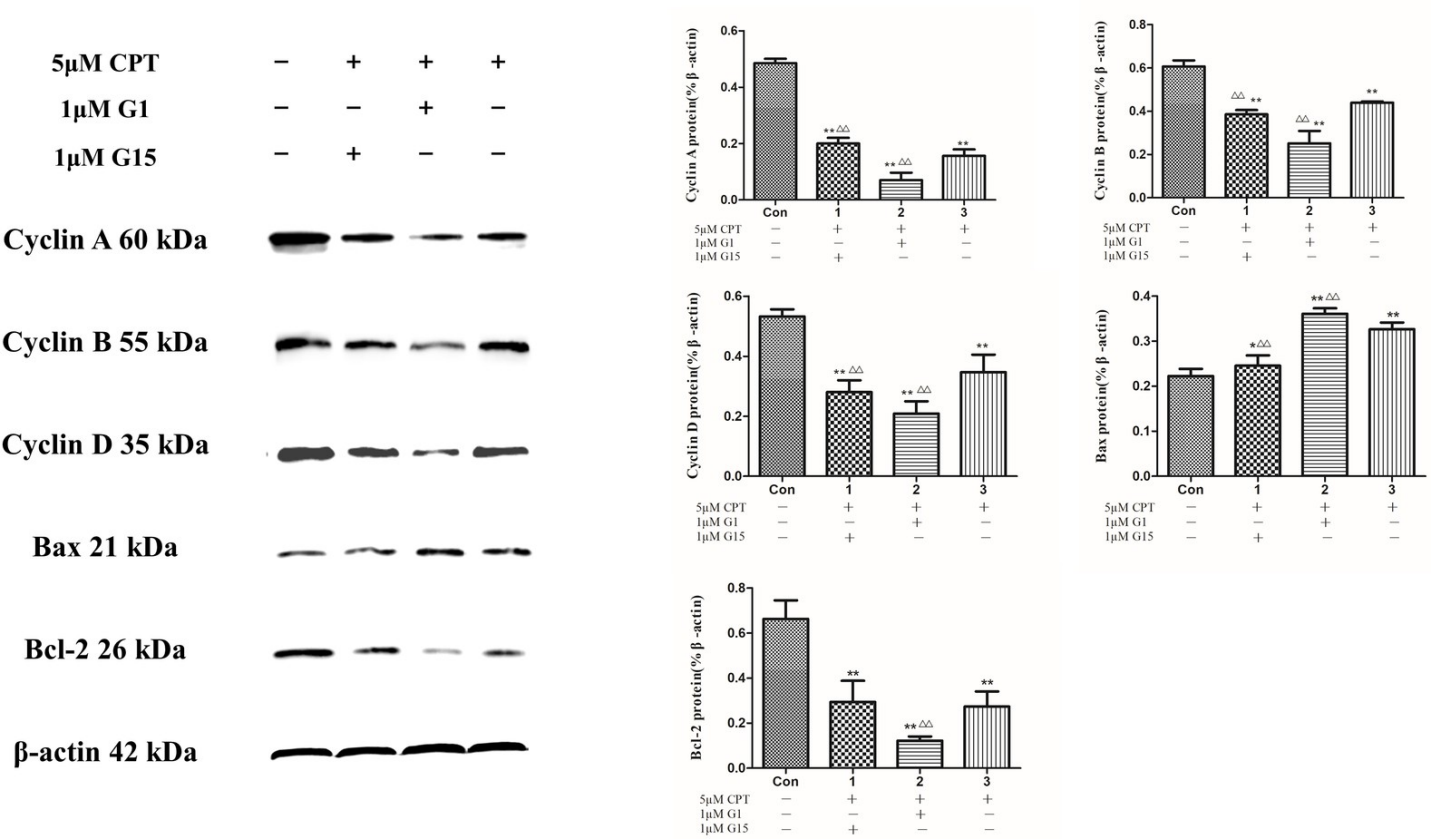
The expression of cell cycle and apoptosis related proteins after treating by G-1 or G-15 together with CPT. The results are expressed as $\bar{x}$ ± S.D. ^**^P < 0.01 or ^*^P < 0.05 compared with control group, ^*△△*^*P* < 0.01 compared with 5 μM CPT group were recognized as statistically significant.

In order to clarify the role of GPER in CPT inducing antiproliferative effect on breast cancer cells, GPER-siRNA transfection was applied to knock down the GPER expression. As shown in Fig 7A, a low expression of GPER in MCF-7 cells was indicated that siRNA technique successfully constucted a cell model with GPER knocked down. And then, a cell proliferation assay was performed on these model cells. The results demonstrated that knockdown of GPER abolishes the decrease in cell viability induced by CPT treatment for 48 h (Fig 7B). Additionally in Fig 8, the expression of cyclins including cyclin D, cyclin B and cyclin A were further decreased after treating by GPER agonist G-1 together with CPT, compared with the reduction of the group treated by antagonist G-15 together with CPT. Consistent with the decreasing of cyclins, the expression of antiapoptotic protein Bcl-2 was also further reducted by treating G-1 together with CPT. On the contrary, the apoptosis related protein Bax was significantly increased after treating by G-1 together with CPT. To summarize, activated GPER might promote the inhibitary effect of CPT on MCF-7 cells.

### PI3K/AKT signaling pathway mediated by GPER might be the molecular mechanism of the anti breast cancer cells effect induced by CPT

**Fig 9:**
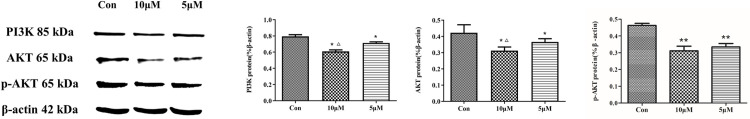
PI3K, AKT and p-AKT were downregulated by CPT in MCF-7 cells. The results are expressed as $\bar{x}$ ± S.D. ^**^P < 0.01 or ^*^P < 0.05 compared with control group and ^*△*^*P* < 0.05 compared with 5 μM CPT group were recognized as statistically significant.

**Fig 10:**
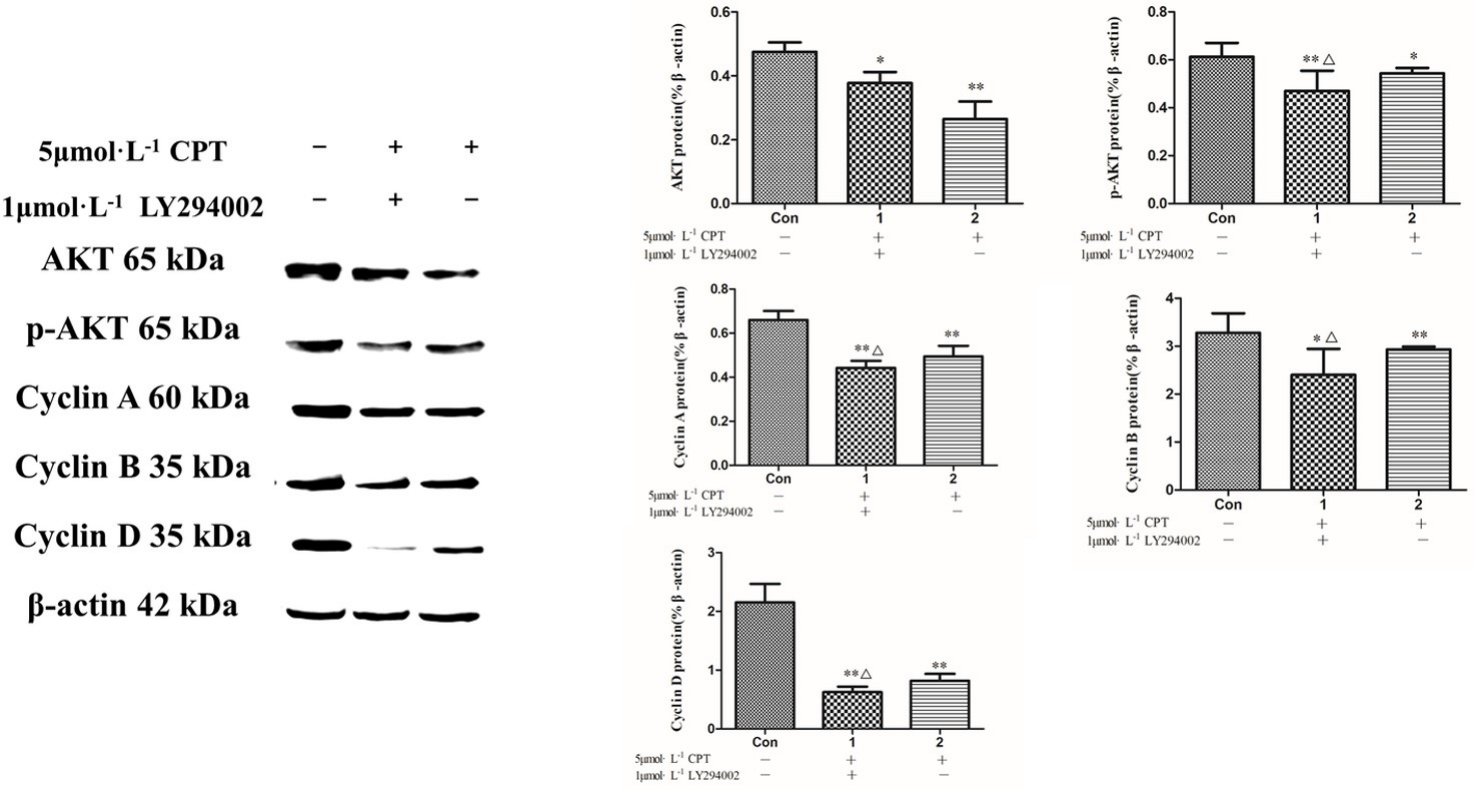
The expression of cyclins and p-AKT after inhibiting PI3K by LY294002. The results are expressed as $\bar{x}$ ± S.D. ^**^P < 0.01 or ^*^P < 0.05 compared with control group, ^*△*^*P* < 0.05 compared with 5 μM CPT group were recognized as statistically significant.

**Fig 11:**
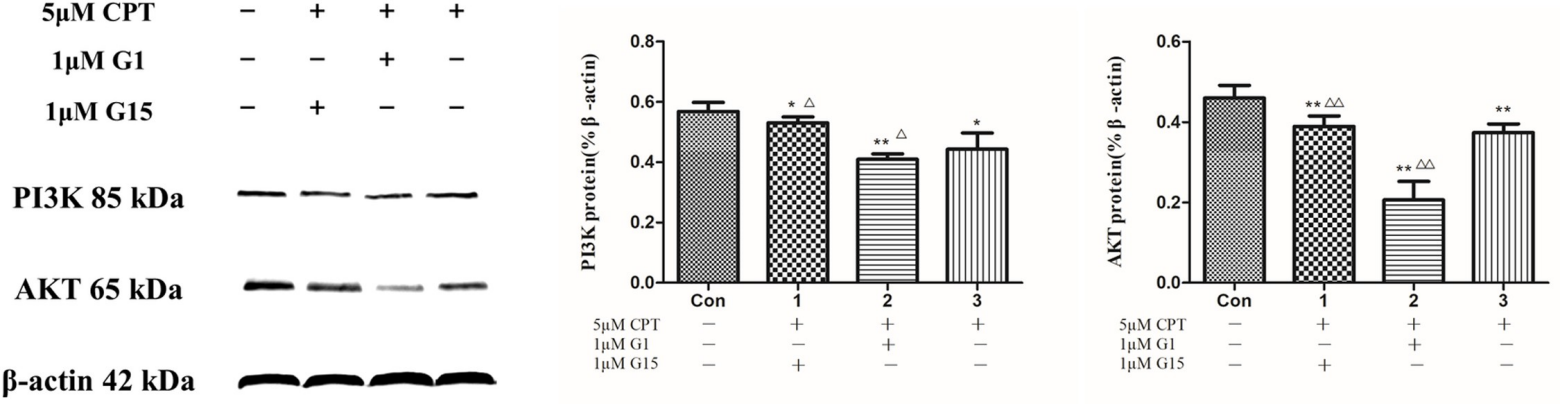
PI3K and AKT expression after treating by CPT with G-1 or G-15. The results are expressed as $\bar{x}$ ± S.D. ^**^P < 0.01 or ^*^P < 0.05 compared with control group, ^*△△*^*P* < 0.01 or ^*△*^*P* < 0.05 compared with 5 μM CPT group were recognized as statistically significant.

In response to CPT for 48 h, a dose and dependent manner reduction of PI3K, AKT and p-AKT expression were detected (Fig 9). To clarify the effects of CPT on the PI3K/AKT signaling and its downstream proteins, a specific PI3K inhibitor-LY294002 was then applied. Treating with LY294002 resulted in an inhibition in expression of AKT, p-AKT and cyclins including cyclin D, cyclin B and cyclin A (Fig 10). Subsequently, G-1 and G-15 were applied to detected the GPER function in this regulation. The rusults indicated that a further reducion of PI3K and AKT expression was significantly presented after treating by G-1 together with 5 μM CPT, however, the decreasing effet was attenuated by using G-15 together with CPT as shown in Fig 11. The findings of the current study clarified that anti breast cancer cells effct of CPT is considered as the inhibition of PI3K/AKT signaling mediated by GPER.

## Discussion

Breast cancer, the most common neoplasm diagnosed among women around world, is one of the leading causes of female cancer death [30]. With the increasing prevelence worldwide, it is necessary to search some new methods natrually. In fact, there has been growing interest in tanshinones, which are the major bioactive compounds of traditional Chinese herb *Savia miltiorrhiza Bunge* roots (also known as Danshen). Danshen has been extensively used for the treatment of various cardiovascular and cerebrovascular diseases [31]. Recently, the effect of Danshen on improving survival of patients with breast cancer aroused researchers’ concern [32]. Especially, kinds of active components extracted from Danshen were confirmed to inhibit the proliferation of breast cancer cells in vitro [33,34]. Cryptotanshinone (CPT), one of the most abundant active compounds in Danshen began to attract much attention on account of its anti inflammatory [35], anti bacterium [36] and anti tumor effects [15,37,38]. Crucially, it has been paid much concern to the anti tumor function and some studies demonstrated that CPT could exert antiproliferative effect and promote apoptosis of breast cancer cells in different pathologic types [39,40]. Nevertheless, the mechanism of its effect still remains vague and imprecise. What is noteworthy is that studies have reported many types of breast cancer cells could express GPER [20,41]. Since the drug resistance in hormone treatment of breast cancer caused by targeting classic estrogen receptors, the discovery of GPER brought a new breakthrough point to illustrate the mechanism of the effects of estrogen or estrogenic substances on proliferation and apoptosis process.

In the current study, we validated that CPT could suppress the MCF-7 cell viability by inducing the G2-phase arrest and cell apoptosis. Importantly, the cell viability inhibition was associated with the expression of GPER. That meant CPT might be recongnized as a novel GPER binding compound. Molecular docking predicted that CPT could target the GPER and has interaction energy comparable to estradiol with GPER. Application of GPER agonist G-1 and antagonist G-15 brought more information to us about the function of GPER regulating the effect of CPT on MCF-7 cells. Concretely, activated GPER could increased the proliferation inhibitary and apoptotic effect of CPT on breast cancer MCF-7 cells.

Cell proliferation was dependent on cell cycle progression. It is of much significance to block the cell cycle in cancer treatment. Therefore, explaining the cell cycle arrest caused by CPT might help us to clarify the molecular mechanism of its anticancer effect. Our study revealed a dose-dependent reduction of the expression of cyclins including cyclin A, cyclin B, and cyclin D after treating by CPT. Notably, the decreasing of cyclin B was relatively weak compared with cyclin A and cyclin D. We have already known that cyclin D, cyclin A and cyclin B play crucial roles in the different progressions of cell proliferation. Concretely, cyclin D was in connection with the progression of G1 to S phase, cyclin A was associated with S to G2 phase and cyclin B took part in the phase from G2 to M [42]. In the previous study, we found a G2/M-phase arrest tested by FCM in MCF-7 after treating by CPT. Consistence with these results, the G2/M-phase related cyclin B could express more than cyclin A and cyclin D due to the cycle arrest.

Furthermore, we observed the apoptosis induced by CPT obviously. Apoptosis is a type of programmed cell death and induction of apoptosis is realized as a key to treat the cancer. Studies have shown that animal cells could exert apoptosis through the similar pathway when received a stimulation by apoptosis-inducing factors, which is dependent on a series of aspartic protease family named caspase [43–45]. Once activated, the primary caspase would cut and activate other caspases in turn to produce a proteolytic cascade of amplification [46]. Among the caspase family, caspase-3 acted as effecor, which took essential part in cutting structural and regulatory proteins in the nuclear and cytoplasm to in- or activate them to ensure the normal process of apoptosis [47,48]. In the current study, the expression of caspase-3 was significantly increased after treating by CPT. It was indicated that the apoptotic process was taking place inside the MCF-7 cells. Additionally, Bcl-2 and Bax belong to the Bcl-2 (the B-cell lymphoma gene 2) family. It has been reported that Bcl-2 could inhibit apoptosis by blocking the release of cytochrome c (cyt c) from mitochondria, however, activation of Bax contributed to the apoptosis [49]. We obseved an increasing expression of Bax and inhibition of Bcl-2 after treatment with CPT. Combined with the apoptosis rate assay tested by FCM, we concluded that CPT could significantly induce the apoptosis of MCF-7 cells.

Furthermore, a number of studies have reported that PI3K/AKT signaling pathway was closely related to proliferation and apoptosis of cancer cells [27,50]. Targeting PI3K/AKT pathway was recognized as a prime strategy in cancer therapy. Consistent with these studies, treated by CPT significantly inhibited the expression of PI3K, AKT, p-AKT and their downstream factors including cyclins and apoptosis related proteins tested in the current study. The specific PI3K inhibitor-LY294002 was used for further clarifying that the inhibition of MCF-7 cell proliferation and induction of apoptosis were regulated by PI3K/AKT signaling pathway. Improtantly, the intervention by G-1 and G-15 gave us more imformation that the effect caused by CPT through PI3K/AKT signal transduction pathway could be mediated by GPER.

## Conclusion

In conclusion, the findings of our current study brought an interesing message that CPT could be a natural molecule against breast cancer MCF-7 cells. Such proliferation inhibatory and apoptotic effects were regulated by GPER and its mediated function on PI3K/AKT signaling pathway. Thus, GPER might be a novel target in breast cancer treatment which is worthy of further study.

## Supporting information

S1 File (RAR)
